# Genome Sequence of *Streptococcus parauberis* Strain KCTC11980, Isolated from Diseased *Paralichthys olivaceus*

**DOI:** 10.1128/genomeA.00780-13

**Published:** 2013-10-03

**Authors:** Myoung Ae Park, Mun Gyeong Kwon, Jee Youn Hwang, Sung Hee Jung, Dong-Wook Kim, Jin-Young Park, Ji-Sun Kim, Yun-Jeong Na, Min-Young Kim, Dae-Soo Kim, Sung-Hwa Chae, Jung Soo Seo

**Affiliations:** Pathology Division, National Fisheries Research and Development Institute (NFRDI), Busan, Republic of Koreaa; Human Derived Material Center, Korea Research Institute of Bioscience and Biotechnology (KRIBB), Daejeon, Republic of Koreab; Research Institute of GnCBIO Co., Ltd., Daejeon, Republic of Koreac

## Abstract

*Streptococcus parauberis* is a coccoid, nonmotile, alpha-hemolytic, Gram-positive bacterium of the *Streptococcaceae* family. *Streptococcus parauberis* strain KCTC11980 was isolated from the kidney of a diseased olive flounder collected from an aquaculture farm on Jeju Island in 2010. The 2.12-Mb genome sequence consists of 44 large contigs in 16 scaffolds and contains 2,214 predicted protein-coding genes, with a G+C content of 35.4%.

## GENOME ANNOUNCEMENT

Streptococcosis mediated by streptococci, such as *Streptococcus iniae* and *S. parauberis*, has been a major cause of mass mortality in marine aquaculture systems (1–3). Among these species, *Streptococcus parauberis* was first described as the etiologic agent of bovine mastitis (4) and later found to cause streptococcosis in cultured turbot (*Scophthalmus maximus*) in Spain (5, 6). Recently, a streptococcal infection caused by *Streptococcus parauberis* has increased in frequency in the olive flounder (*Paralichthys olivaceus*) aquaculture industry in South Korea (7, 8). *S. parauberis* is a coccoid, nonmotile, alpha-hemolytic, Gram-positive bacterium of the *Streptococcaceae* family. *S. parauberis* and *S. uberis* are common agents of bovine mastitis (4). Genomic DNA was extracted from cultured bacterial isolates using the alkaline lysis method (9). We sequenced the genome of *S. parauberis* because it had not been sequenced at the time our sequencing project began, according to the Genomes OnLine Database (GOLD) (10). We report the genome sequence of *S. parauberis* strain KCTC1980, obtained using a whole-genome shotgun strategy (11) with a Roche 454 GS (FLX titanium) pyrosequencing system (775,572 reads, totaling ~113.6 Mb; ~28.9-fold coverage of the genome). Pyrosequencing was processed using Roche’s software, according to the manufacturer’s instructions. All of the paired reads were assembled using the Newbler assembler 2.6 (454 Life Science), which generated 66 contigs (GenBank accession no. BANN01000001 through BANN01000066), and the assembly falls into 16 scaffolds (accession no. DF266806 through DF266821). The predicted proteins were annotated using the Basic Local Alignment Search Tool (BLAST) (12) and the Rapid Annotation using Subsystem Technology (RAST) server (13). In addition, open reading frame (ORF) prediction was performed using CD-HIT software, with which contigs were searched against the Glimmer 3.02 modeling software package and GeneMark version 2.5 (14), tRNAscan-SE 1.21 (15), RNAmmer 1.2 (16), and Clusters of Orthologous Groups (COG) (17) databases to annotate the gene descriptions.

The *S. parauberis* draft genome includes 2,127,951 bp and is composed of 2,214 predicted coding sequences (CDS), with a G+C content of 35.4%. The genome contained representatives of 311 subsystems, and we used this information to reconstruct the metabolic network (determined using the RAST server). A distinguishing subsystem feature was the absence of genes corresponding to a conserved domain protein, ABC transporter, ATP-binding protein, membrane protein, and transcriptional regulator. The CDS annotated by the COG database were classified into eight major categories (R, G, S, K, J, E, L, and P) from among the 42 COG groups. The enzymes identified included pantothenate kinase (EC 2.7.1.33), tRNA pseudouridine synthase A (EC 5.4.99.12), and DNA polymerase III subunit delta (EC 2.7.7.7). More detailed analysis of this genome and a comparative analysis with other *S. parauberis* genomes may well identify other genes upon finalization of the genome.

### Nucleotide sequence accession numbers.

The draft genome sequence of *S. parauberis* KCTC11980 is available in GenBank under the accession number BANN00000000 (DF266806 through DF266821). The version described in this paper is version BANN01000000.
